# Positive Charges in the Brace Region Facilitate the Membrane Disruption of MLKL-NTR in Necroptosis

**DOI:** 10.3390/molecules26175194

**Published:** 2021-08-27

**Authors:** Yaqing Yang, Encheng Xie, Lingyu Du, Yu Yang, Bin Wu, Liming Sun, Shuqing Wang, Bo OuYang

**Affiliations:** 1 State Key Laboratory of Molecular Biology, Shanghai Institute of Biochemistry and Cell Biology, CAS Center for Excellence in Molecular Cell Science, Chinese Academy of Sciences, Shanghai 200031, China; yangyaqing2015@sibcb.ac.cn (Y.Y.); xieencheng2019@sibcb.ac.cn (E.X.); dulingyu@sibcb.ac.cn (L.D.); yangyu2014@sibcb.ac.cn (Y.Y.); liming.sun@sibcb.ac.cn (L.S.); 2 University of Chinese Academy of Sciences, Beijing 100049, China; 3 National Facility for Protein Science in Shanghai, ZhangJiang Lab, Shanghai Advanced Research Institute, Chinese Academy of Sciences, Shanghai 201203, China; bin.wu@sibcb.ac.cn; 4 School of Pharmacy, Tianjin Medical University, Tianjin 300070, China

**Keywords:** MLKL, brace helix, auto-inhibitory, MD simulation

## Abstract

Necroptosis is a type of programmed cell death executed through the plasma membrane disruption by mixed lineage kinase domain-like protein (MLKL). Previous studies have revealed that an N-terminal four-helix bundle domain (NBD) of MLKL is the executioner domain for the membrane permeabilization, which is auto-inhibited by the first brace helix (H6). After necroptosis initiation, this inhibitory brace helix detaches and the NBD can integrate into the membrane, and hence leads to necroptotic cell death. However, how the NBD is released and induces membrane rupture is poorly understood. Here, we reconstituted MLKL_2__–154_ into membrane mimetic bicelles and observed the structure disruption and membrane release of the first brace helix that is regulated by negatively charged phospholipids in a dose-dependent manner. Using molecular dynamics simulation we found that the brace region in an isolated, auto-inhibited MLKL_2__–154_ becomes intrinsically disordered in solution after 7 ns dynamic motion. Further investigations demonstrated that a cluster of arginines in the C-terminus of MLKL_2__–154_ is important for the molecular conformational switch. Functional mutagenesis showed that mutating these arginines to glutamates hindered the membrane disruption of full-length MLKL and thus inhibited the necroptotic cell death. These findings suggest that the brace helix also plays an active role in MLKL regulation, rather than an auto-inhibitory domain.

## 1. Introduction

Necroptosis is a type of programmed necrosis that has been implicated in host immune defense and various human diseases [1,2]. Mixed lineage kinase domain-like protein (MLKL) is the terminal key mediator in the necroptotic pathway discovered by Sun et al. in 2012 [3]. In healthy cells, MLKL is found in the cytosol as a monomeric pseudokinase [4]. Upon activation, MLKL is phosphorylated by Receptor Interacting Protein Kinase 3 (RIPK3) [5], which drives MLKL oligomerization and translocation from the cytosol to the plasma membrane and leads to cell membrane disruption. In the past decade, a great deal of extensive studies on MLKL have been conducted [6,7]. However, the precise mechanism of MLKL activation, including the oligomerization, membrane translocation and permeabilization, is still under debate.

MLKL oligomerization is an essential step in the membrane disruption [8]. Active MLKL is thought to form trimers [9], tetramers [10], hexamers [5] and octamers [11]. Liu et al. also reported that the MLKL tetramers can further polymerize to form disulfide bond-dependent amyloid-like fibers [12]. The cryo-EM structure of MLKL from *Arabidopsis* (*At*) determined recently showed a tetrameric assembly with a similar monomer structure to vertebrate MLKLs [13]. However, the N-terminal HeLo domain in *At*MLKL is sequestered, indicating this plant tetrameric assembly is an inactive form. The respective oligomerization for MLKLs in the active states may be quite distinct. Previous studies have revealed that negatively charged lipids play an important role in MLKL activation, allowing MLKL to translocate and become incorporated into the plasma membrane. Phosphatidylinositol phosphates and cardiolipin interacting with the positively charged amino acids on MLKL serve as critical binders of MLKL in necroptosis [14]. Moreover, the combined effects of inositol phosphate metabolites IP4, IP5, and IP6 were demonstrated to be essential for necroptotic induction by human MLKL [15,16]. However, alanine substitution of acidic and aliphatic residues also perturbs cell killing [17,18,19], suggesting that interactions other than electrostatic interactions with negatively charged phospholipids attribute to membrane association.

The pore-formation and lipid-binding ability reside in an N-terminal four-helix bundle domain (NBD, amino acid residues 1–124 in human) of MLKL [20], which is connected by a two-helix brace to a C-terminal pseudokinase domain (psKD) (Figure S1A) [21,22]. The psKD containing the phosphorylation site mediated by RIPK3 serves as a receiver for upstream signals [3]. The NBD of MLKL acts as the executioner domain that NBD alone can disrupt the membrane, and mutations on the NBD lose the ability to promote MLKL necroptosis [14]. A solution structure of the MLKL N-terminal region (NTR) determined by NMR spectroscopy showed that the NBD is auto-inhibited by the first brace helix (H6) and its preceding linker [20]. Upon necroptosis initiation, the auto-inhibitory region is released from the NBD and then the four-helix bundle is exposed to oligomerize and integrate into the plasma membrane, which eventually leads to cell death. A recent study further showed that the first brace helix not only acts as a plug to prevent opening of the bundle, but also interacts with the second brace helix and contributes to MLKL conformational changes and oligomerization [23].

So far, the crystal structures of the human MLKL psKD [22] and full-length mouse MLKL [22], together with the NMR structure of MLKL-NTR [20] provided a clear view of how these domains are arranged in solution. However, the structure visualization of the NTR in a lipid environment is still lacking. Here, we sought to shed light on the membrane-associated architecture of MLKL-NTR. Reconstitution of the NTR of MLKL in membrane mimetic bicelles showed the release of auto-inhibitory region regulated by negatively charged phospholipids in a dose-dependent manner. Using molecular dynamics (MD) simulation, we found the intrinsic disordered nature of the first brace helix. Furthermore, an arginine cluster in the C-terminus of the NTR was revealed to facilitate the release of the auto-inhibitory region, and mutating these basic residues significantly inhibited MLKL activity. Our results suggest that the first brace helix is more than an auto-inhibitory domain in MLKL-dependent necroptosis.

## 2. Results

### 2.1. MLKL_2__–154_ Reconstituted into Bicelles

Previously, Su et al. used nanodiscs and liposomes to study how MLKL_2__–154_ binds to membranes by NMR spectroscopy, in which the addition of MLKL_2__–154_ to nanodiscs containing 15% cardiolipin did not affect the cross-peak intensities of the two dimensional (2D) ^1^H-^15^N TROSY-HSQC spectrum, while the addition of MLKL_2__–154_ to liposomes with 15% cardiolipin significantly decreased the peak intensities [20]. The obstacles to observe MLKL_2__–154_ in these two membrane systems are due to nanodiscs that are restricted by the scaffolding protein to make enough room for the MLKL_2–154_ to insert into the bilayers, while the size of liposomes are too large for NMR studies. Detergent micelles have been used to monitor MLKL-NTR binding to inositol phosphates [15,24] and phosphatidylinositols [25], but no details about the membrane insertion of MLKL. Moreover, detergents form monolayer in micelles which do not provide a bilayer membrane mimetic environment. To study the membrane-associated MLKL-NTR, we chose bicelles, which form discoid-shaped phospholipid bilayers composed of long-chain and short-chain phospholipids and give a more native-like membrane system amenable to solution NMR technology.

**Figure 1:**
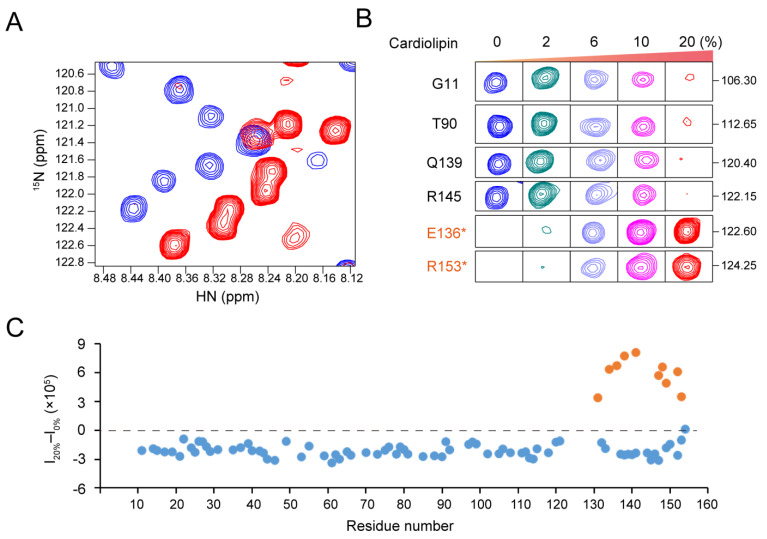
Negatively charged lipids promote the membrane-association of MLKL_2–154_. (**A**) The overlaid 2D ^1^H-^15^N TROSY-HSQC spectra of MLKL_2–154_ in DMPC bicelles with (red) and without (blue) 20% cardiolipin. (**B**) The peak of six residues of MLKL_2__–154_ in DMPC bicelles supplemented with different concentrations of cardiolipin. The first left panel shows the same spectral region as the right panels for each residue. Panels 2–5 are spectra-recorded at increasing concentrations of 2%, 6%, 10%, and 20% cardiolipin. Residue names marked by an asterisk and colored in orange are new resonances which appeared upon the addition of cardiolipin. (**C**) Peak intensity changes (I_20%_–I_0%_) between MLKL_2–154_ in DMPC bicelles with (I_20%_) and without (I_0%_) 20% cardiolipin. The orange dots denote the newly emerged resonances. The assignment of resonance peaks was conducted as described below.

Purified human MLKL_2–154_ was first reconstituted into bicelles composed of 1,2-dimyristoyl-sn-glycero-3-phosphocholine (DMPC), and 1,2-dihexanoyl-sn-glycero-3-phosphocholine (DHPC) with a q value (DMPC:DHPC, molar ratio) of about 0.6. The 2D ^1^H-^15^N TROSY-HSQC spectrum of MLKL_2–154_ (0.1 mM) reconstituted in DMPC bicelles showed very little chemical shift differences from the spectrum of MLKL_2–154_ in solution and the peak intensities barely changed (Figure S1B–D). However, a new set of resonances of MLKL_2–154_ appeared in DMPC bicelles at high concentrations (Figure S1E,F), indicating that MLKL-NTR has the ability to interact with the membrane in the presence of DMPC only. Earlier work showed that MLKL can localize to membranes throughout the cell, including mitochondrial membranes [5,26,27]. MLKL can also bind to phosphoinositides and cardiolipin [5,14], and permeabilize cardiolipin-containing liposomes in vitro [5,20]. DMPG (1,2-dimyristoyl-sn-glycero-3-phosphoglycerol) is a widely used negatively charged phospholipid in NMR to mimic PGs in the natural membrane. Therefore, we supplemented the bicelles with different concentrations of cardiolipin (Figure 1) and DMPG (Figure S2) to observe the effects of negatively charged lipids on MLKL. Increasing the amount of cardiolipin and DMPG, the intensities of most of the resonances decreased significantly (Figure 1 and Figure S2), while the new set of resonances was detected with significantly increasing intensities (Figure 1 and Figure S2). These results suggested that negatively charged lipids promote membrane-association of MLKL_2–154_, which is consistent with the previous studies.

### 2.2. Negatively Charged Phospholipids Induced the Release of the Brace Region

**Figure 2:**
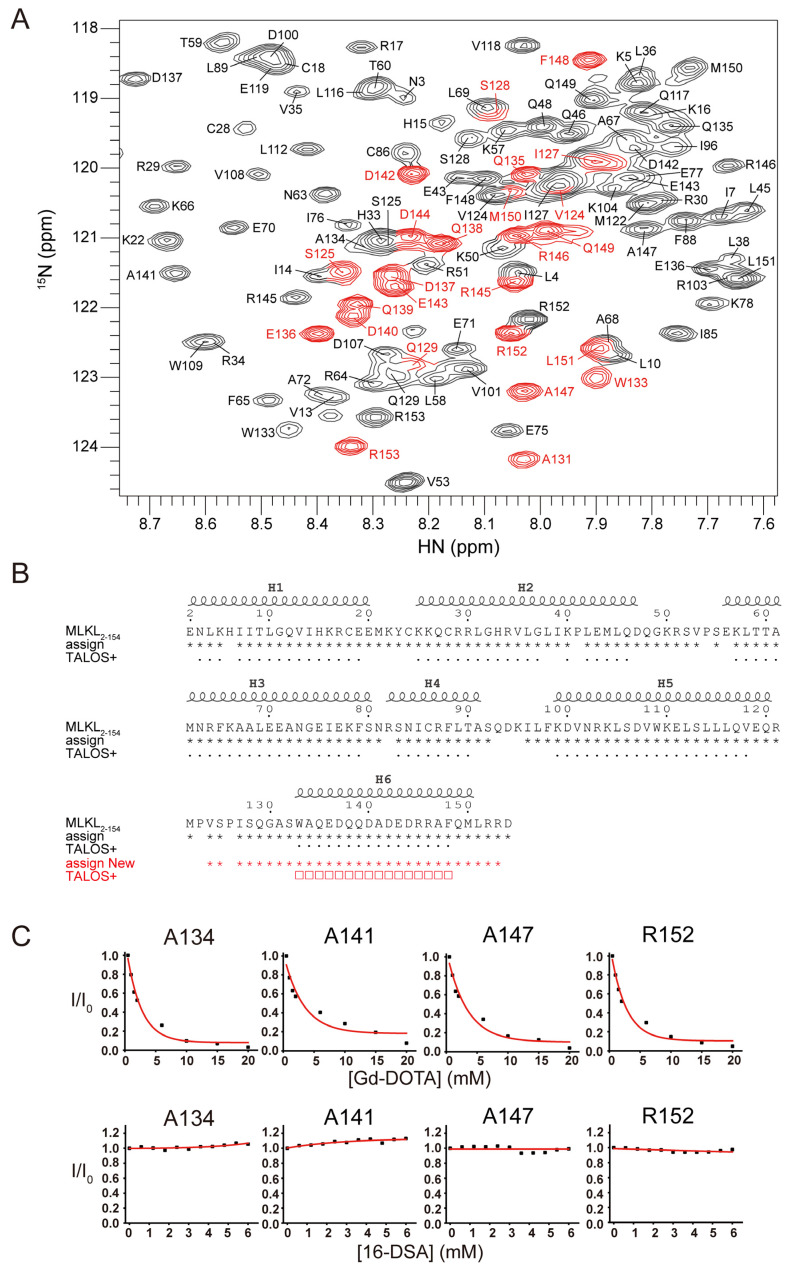
The distortion and the release of the auto-inhibitory region of MLKL_2–154_. (**A**) Backbone assignment of 0.6-mM *U*-[^15^N, ^13^C, ^2^H] MLKL_2–154_ in DMPC bicelles with 10% cardiolipin. The resonances in red are the new set of resonances. (**B**) Primary and secondary structure alignment of MLKL_2–154_ in DMPC bicelles with 10% cardiolipin. The coils above the sequence indicate the helical regions of MLKL_2–154_ NMR structure (PDB code: 2MSV). Asterisks (*) below the sequence indicate the MLKL_2–154_ residues with NMR chemical-shift assignment and dots (·) represent helical regions of MLKL_2–154_ predicted by TALOS+ based on chemical-shift values. The new set of resonances were colored in red and marked by squares (□). (**C**) Residue-specific PREs were measured by recording 2D ^1^H-^15^N TROSY-HSQC spectra when titrated with water-soluble paramagnetic agent Gd-DOTA (upper panel) and membrane-embedded paramagnetic agent 16-DSA (lower panel). I/I_0_ vs. Gd-DOTA or 16-DSA concentrations were plotted for each residue, in which I and I_0_ are the intensity of a peak in the presence and absence of the paramagnetic agent, respectively.

To further identify the appeared new resonances, we prepared a 0.6 mM *U*-[^15^N, ^13^C, ^2^H] MLKL_2–154_ sample in bicelles supplemented with 10% cardiolipin. 96% of the backbone resonances of non-proline residues of MLKL_2–154_ were assigned using a combination of triple-resonance and NOE experiments. The new set of resonances belong to residues V124–R153 (Figure 2A), i.e., H6 and its preceding linker, indicating membrane-associated MLKL_2–154_ presented a different conformation on this region. The backbone ^13^C^α^, ^13^C^β^, and ^13^C’ chemical shifts analyzed by TALOS+ program showed that the membrane-associated new resonances are disordered (Figure 2B), indicating the addition of negatively charged phospholipids induced the unfolding of a helical region (W133–M150). Paramagnetic relaxation enhancement (PRE) approaches using the water-soluble paramagnetic agent gadolinium(III) 1,4,7,10-tetraazacyclododecane-1,4,7,10-tetraacetate (Gd-DOTA) and membrane-embedded paramagnetic agent 16-doxyl-stearic acid (16-DSA) further confirmed the disordered bracelet in MLKL_2–154_ is solvent-exposed in DMPC bicelles with 10% cardiolipin (Figure 2C). These results are consistent with previous studies that negatively charged phospholipids induced the distortion and the release of the auto-inhibitory region of MLKL [15,16,25]. On the other hand, the membrane-associated NBD cross-peaks were broadened beyond detection. Such line-broadenings on NBD are either caused by the direct membrane insertion or the oligomer formation upon membrane interaction.

### 2.3. The Intrinsic Disordered Properties of H6 Helix

We next performed the molecular dynamics (MD) simulation to characterize local changes along the protein chain. As we mentioned above, a bulk of studies revealed that the NTR alone can permeabilize liposomes and induce necroptosis in different cell lines, while the pseudokinase domain is not required for the membrane permeabilization [5,17,20,27]. We only used MLKL-NTR for the MD simulation. The NMR structure of MLKL_2–154_ (PDB code: 2MSV) was first embedded in a periodic orthorhombic box (~20 × 20 × 20 Å^3^) containing the explicit simple point charge (SPC) water molecules. MD simulations were carried out as described in Materials and Methods. 100 ns of NPT production simulation without any restriction were first run and recorded. The Root Mean Square Fluctuation (RMSF) of the backbone and heavy atoms showed that H6 fluctuates most of the time during the simulation, indicating that the helix structure of H6 is not rigid in the entire simulation.

**Figure 3:**
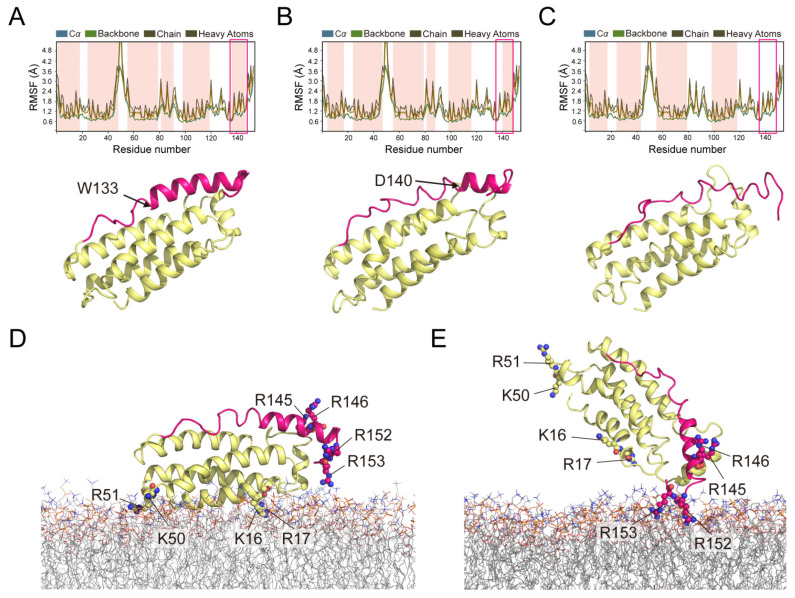
The MD simulations of MLKL_2–154_ in pure water and in membranes. (**A**) The starting protein structure for 2MSV. The H6 helix (from residues 133 to 150) is present at that point in time and lasted for the first 7 nanoseconds. The RMSF is used for characterizing local changes along the protein chain, along with SSE (Secondary Structure Elements) of MLKL_2__–154_ highlighted in which α-helical regions are highlighted in pink. (**B**) The average structure over the 8th nanosecond, the residues 133–139 are no longer the SSE composition. (**C**) Over the entire simulation time (100 ns), H6 helix is missing, however, the helical structures of the NBD are still stable. (**D**) The initial position of MLKL_2__–154_ (PDB code: 2MSV) on the DMPC membrane predicted by the PPM Server. K16, R17, K50 and R51 are engaged initially opposite to the brace. (**E**) Over 100 ns simulation time, the positively charged cluster in the auto-inhibitory region got close to the membrane with R152 and R153 inserting into the membrane.

We extracted the average structures according to 1 ns interval from the 100 ns MD trajectory files, a total of 100 average structures were exported. The helical structure of H6 is stable at the first 7 ns (Figure 3A). Starting from 8 ns, we found that H6 began to uncoil its helical structure on amino acids 133–139 (Figure 3B). At the end of 100 ns, the whole helix structure of H6 is disrupted (Figure 3C). Subsequently, we carried out an extended period of dynamics simulation (100 ns to 1000 ns), and the uncoiled H6 maintained its disordered state without any helical structure recovered. No α-helical structure of the residues in the first brace helix is kept at the end of 1000 ns MLKL_2–154_ simulations. While the four helix bundle remained its stable secondary structure during the simulation.

We further put the protein system into the membrane system for dynamic simulation. The NMR structure of the protein (PDB code: 2MSV) was initially placed on the membrane (DMPC) according to a previously proposed position by Quarato et al. [25] (Figure 3D) via the module system builder in Desmond software. DPPC, POPC, POPE and DMPC are the only available lipids to build the model membrane, no negatively charged lipids are available due to the technical limitations of the software. As shown above, MLKL_2–154_ can interact with DMPC bicelles at high concentrations; thus, MD simulation in DMPC can still offer us some worthy hints to inspect the membrane-association of MLKL.

The predicted composite structure showed that H1 and H2 is close to the membrane surface (Figure 3D), while H6 is opposite to the membrane binding sites. Then this predicted composite structure, protein and membrane, was embedded in a periodic orthorhombic box (~40 × 40 × 40 Å^3^) containing the SPC water molecules. After 100 ns MD simulation, we found that the helical structure of H6 did not uncoil and become an irregular structure like in the pure water environment. Surprisingly, H6 is gradually getting close to the membrane surface and R152 and R153 inserted deeply into the membrane after 100 ns simulation (Figure 3E). However, further MD simulation for the protein-membrane system did not show how the protein system enter the membrane and disrupt the membrane structure.

### 2.4. Polybasic Residues in the C-Terminus of the Brace Region Is Essential for the Release

The last snapshot from the apo MLKL simulation showed that the unfolded H6 attaches to the NBD with the salt bridges between four basic residues (K26, R30, R34 and K95) of NBD and four acidic residues (E136 and D140, D142, E143) of H6 (Figure S3A), and hydrophobic interactions were also kept at three different sites, including residues L38, P41, M44, L45, L89, L97, L116, M122, V124, I127, A141 and F148 (Figure S3B), indicating that the H6 is still locked in the inhibitory conformation even its structure is disrupted. While MD simulations in the membrane environment showed a cluster of basic charged arginines in C-terminus of the auto-inhibitory region (R152 and R153) (Figure S4A) interacts with the membrane, along with that R145 and R146 move very closely to the membrane. These basic residues are also highly conserved in primate species (Figure S4B); therefore, we guess that their interactions with the membrane may provide the extra pulling-away forces to facilitate the release of the auto-inhibited region.

**Figure 4:**
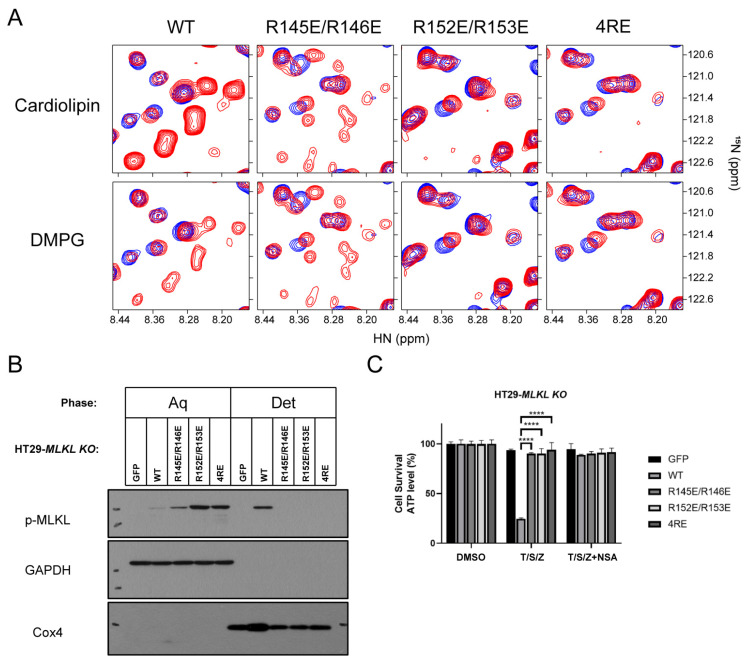
The arginine cluster acts as a critical element for the release of the auto-inhibitory region. (**A**) Superimposed 2D ^1^H-^15^N TROSY-HSQC spectra of WT MLKL_2–154_ and mutants (R145E/R146E, R152E/R153E, 4RE) in solution (blue) and in DMPC bicelles with 10% cardiolipin or DMPG (red), respectively. (**B**) MLKL positive mutants cannot be concentrated in membrane fraction after phase separation. HT29-MLKL KO were lentvirally reconstituted with different MLKL mutants. Cells were treated with T/S/Z for 6 h. T/S/Z treatment indicates TNF (20 ng/mL), Smac (100 nM), zVAD (20 μM). Results are reported from one representative experiment from at least three independent repeats. (**C**) MLKL mutants suppressed T/S/Z induced necroptosis. HT29-MLKL KO cell line was treated with T/S/Z for 10 h. T/S/Z treatment indicates TNF (20 ng/mL), Smac (100 nM), zVAD (20 μM). *p* values were determined by unpaired two-tailed Student’s *t*-test with Welch’s correction. **** *p* < 0.0001. All results are reported from one representative experiment from at least three independent repeats.

To investigate this hypothesis, we first mutated these arginine residues to glutamate to eliminate the possible electronic interactions between the lipids and the basic region. Three different mutants were constructed, including R145E/R146E, R152E/R153E and R145E/R146E/R152E/R153E (4RE). The 2D ^1^H-^15^N TROSY-HSQC experiments showed that inverting the charge of these amino acids did not interfere with the overall MLKL_2–154_ structure (Figure S4C). Adding 10% cardiolipin or DMPG to the reconstituted mutants in DMPC bicelles did not increase the intensities of the new sets of disordered H6 for the R152E/153E and 4RE mutants, nor decreased the intensities of other resonances (Figure 4A). The R145E/R146E mutant also showed similar changes but to a lesser extent (Figure 4A).

We further performed a phase separation assay—Triton X-114 assay—to investigate the effects of these polybasic residues on MLKL membrane translocation following previous protocols. The three full-length MLKL mutants R145E/R146E, R152E/R153E and 4RE were constructed into HT29 cells via lentivirus transfection. The cells were then harvested after T/S/Z treatment and extracted by Triton X-114 lysis buffer (Figure 4B). At high temperatures, phosphorylated MLKL was concentrated in the detergent phase fraction (the membrane fraction). The Western blotting analysis using antibodies showed that only wild-type MLKL appeared in the detergent phase during necroptosis (Figure 4B). MLKL mutants were not able to transit to the detergent phase, indicating that these mutants did not translocate to the membrane fraction upon necroptosis activation. Cell death was also monitored for different MLKL mutant cell lines. When expressed in cells, MLKL mutants can no longer induce necroptosis after T/S/Z treatment, though they were expressed at similar or much higher levels than the wild-type lethal counterparts (Figure 4C).

Together, these results indicated that the positively charged cluster at the C-terminus of NTR is important to unleash the auto-inhibitory plug from the four-helix bundle.

## 3. Discussion

The structural characterization of the membrane-associated MLKL is a long standing question in the necroptosis studies. Previous solution-state NMR studies have been applied to MLKL-NTR reconstituted in liposomes, nanodiscs [20] and micelles [15,24,25]. In the present study, we reconstituted MLKL_2__–154_ into bicelles, a lipid-bilayer system with better flexibility than nanodiscs and a smaller size than liposomes, to investigate the activation of NTR. Unfortunately, this system did not provide further structural information about how MLKL-NTR inserts into the membrane. Consistent with previous studies, the activation of MLKL-NTR was featured by an unfolding of the first brace helix and detachment of this disordered region [15,16,25], in our study, starting with residue 124 and stretching to residue 153. The release of the auto-inhibitory region was observed upon the addition of negatively charged lipids in a dose-dependent manner. These results confirmed the speculation that the negatively charged phospholipids promote the release of the auto-inhibitory region.

Further investigation of membrane association of MLKL-NTR by MD simulation showed the dynamic property of the auto-inhibitory region. H6 began to unfold its helical structure after 7 ns simulation time in solution and the uncoiled H6 did not recover its secondary structure after 1000 ns dynamics simulation. This result indicated the intrinsic-disordered property of the auto-inhibitory brace. However, during the simulation the auto-inhibitory brace stably attached to the NBD, with the maintenance of hydrophobic interactions between the brace, its preceding linker and the NBD, the electrostatic interactions between the NBD and the brace. Our MD simulation showed the average α-helical percentage of the first brace helix is close to 0% in apo MLKL_2–154_ simulations, which is lower than that (35%) from the simulations performed by Bansal et al. using the full-length model of human MLKL [28]. The construct used in our study only contains the first brace helix, so the unfolding is not restricted by the second brace helix.

The MD simulation of MLKL_2–154_ in DMPC lipids suggested that the positive cluster in the C-terminus interacts with the membrane. Protein-membrane interaction is universally regulated by electrostatic interactions between the negatively charged phospholipids and the positively charged residues of the membrane-binding protein [29]. Previous studies have demonstrated that the interaction of negatively charged phospholipids with the clusters of positively charged residues in the NBD led to the displacement of the first brace [15,16,20,25]. Here, we identified that a cluster of positively charged arginines in C-terminus of the auto-inhibitory region (R145, R146, R152 and R153) also facilitated the unleashing of the brace and the linker during activation. We further confirmed their role by the functional mutagenesis that mutations of arginines to glutamates significantly blocked the necroptosis of MLKL. We noticed that the mutations also affected the phosphorylation levels; however, the phosphorylated mutants enriched in the aqueous fraction instead of the membrane fraction, indicating that these residues are important for the membrane association. It is likely that the arginine cluster interacts with the membrane and pulls the auto-inhibitory plug away from the necrotic NBD; therefore, the four-helix bundle can expose its hydrophobic interior to favor the membrane insertion. Further investigations are required to fully understand the role of this basic cluster in multiple steps of the membrane disruption.

H6 in the auto-inhibitory region was previously reported to contribute to the membrane insertion and oligomerization by interacting with the second brace helix [23], indicating an active role of the auto-inhibitory region. Our study also showed a new role of the auto-inhibitory region that is not passive in the membrane disruption, which provides a better understanding of the activation steps on MLKL recruited to the plasma membrane.

## 4. Materials and Methods

### 4.1. Protein Expression and Purification

Human MLKL_2–154_ was cloned into pET28a(+) vector with an N-terminal 8× His tag followed by a human rhinovirus 3C protease recognition sequence (LEVLFQGP). The recombinant plasmid was transformed into an *E. coli* BL21(DE3) strain. The cells were grown at 37 °C in M9 minimal media until OD_600_ reached 0.8–1.0, then treated with 0.5 mM IPTG and grown at 18 °C. Cells were harvested after 18 h induction and resuspended with buffer A (50 mM HEPES/pH7.5, 300 mM NaCl, 0.2 mM β-ME). Cells were disrupted with a high pressure homogenizer. After cell disruption, soluble part was collected by centrifuging at 40,000× *g* for 30 min and purified by immobilized metal affinity chromatography using an Ni-NTA column. The column was first washed using buffer A with 20 and 40 mM imidazole respectively after sample loading, then eluted by buffer A with 400 mM imidazole. The sample was then dialyzed against buffer A in 3.5 KDa MWCO SnakeSkin dialysis tubing (Thermo Scientific, Rockford, IL, USA) to remove imidazole. The protein was incubated with 3C protease at a molar ratio of 20:1 at 4 °C overnight and applied to an Ni-NTA column to remove 8× His tag. The sample was further purified by size exclusion chromatography using HiLoad 16/600 Superdex 200 pg column (GE) with buffer B (20 mM MES/pH 6.5, 100 mM NaCl). MLKL_2–154_ is easily oxidized, thus reducing agents are required in the final NMR sample. A typical NMR sample consists of 0.1–0.6 mM MLKL_2__–154_ in NMR buffer (20 mM MES/pH 6.5, 100 mM NaCl, 20 mM DTT). The mutants R145E/R146E, R152E/R153E and 4RE were expressed and purified following the same procedure as for the WT.

### 4.2. Reconstitution of MLKL_2–154_ into Bicelles

To prepare empty bicelles, 6.78 mg DMPC (and 0.75 mg cardiolipin or DMPG if needed) was dissolved in CH_3_Cl and dried in a glass tube under nitrogen gas until a thin lipid film was formed. After lyophilization, the lipids were resuspended in 500 μL NMR buffer containing 9.07 mg DHPC. The mixture was vortexed, frozen in liquid nitrogen and thawed at 37 °C for at least 5 times. A 0.1 mM NMR sample was made by incubating the empty bicelles with 50 nmol MLKL_2–154_ for 30 min at room temperature and concentrating the protein-bicelles mixture to 500 μL. The bicelle q value of the NMR sample was carefully controlled at ~0.6 according to 1D ^1^H NMR spectrum. The mutants R145E/R146E, R152E/R153E and 4RE were reconstituted into bicelles with the same protocol as for the WT.

### 4.3. Titration of Negatively Charged Phospholipids

NMR samples for titration experiments contained 0.1 mM MLKL_2__–154_ reconstituted into bicelles supplemented with negatively charged cardiolipin (0–20%) or DMPG (0–20%). The percentage denotes the mass ratio between negatively charged lipids and total lipids (negatively charged lipids + DMPC) in the lipid bilayer. Intensities of backbone NH resonances were recorded using 2D ^1^H-^15^N TROSY-HSQC experiments.

### 4.4. NMR Resonance Assignment and Secondary Structure Prediction

3D TROSY-based HNCA, HN(CO)CA, HN(CA)CO, and HNCO experiments were performed for sequence-specific assignment of backbone chemical shifts (^1^H^N^, ^15^N, ^13^C^α^, and ^13^C’). The assignments were validated by a 3D (H^N^,H^N^)-HSQC-NOESY-TROSY spectrum (150 ms NOE mixing time) and a 3D TROSY-based HN(CO)CACB spectrum. All these experiments were conducted at 30 °C on a 600 MHz Bruker spectrometer equipped with a cryogenic TXI probe. A 0.6-mM *U*-[^15^N, ^13^C, ^2^H] MLKL_2–154_ protein in DMPC bicelles with 10% cardiolipin was used for these experiments. The NMR spectra were processed with NMRPipe and analyzed using XEASY and CcpNmr. Secondary structures of MLKL_2–154_ reconstituted in DPMC bicelles with 10% cardiolipin were predicted based on secondary chemical shift using TALOS+.

### 4.5. Solvent PRE Analysis

Gd-DOTA, a water-soluble and membrane-inaccessible paramagnetic agent, was used to probe membrane immersion depth of protein in bicelles as described by Piai et al. [30]. 0.6-mM *U*-[^15^N, ^2^H] MLKL_2–154_ was reconstituted in DMPC bicelles with 10% cardiolipin and Gd-DOTA was titrated outside the bicelles at the concentrations of 0, 1, 1.5, 2, 6, 10, 15, 20 mM respectively. Peak intensities in the presence (I) and absence (I_0_) of Gd-DOTA were recorded by 2D ^1^H-^15^N TROSY-HSQC spectra (3.5 s recover delay) using a 700 MHz Agilent spectrometer during titration. I/I_0_ vs. Gd-DOTA concentration was plotted for each assigned residue.

### 4.6. Lipophilic PRE Analysis

To further determine the membrane association of MLKL_2–154_, a membrane-embedded paramagnetic agent 16-DSA was titrated to the MLKL_2–154_ sample. The stock solution for titrations was composed of 20 mM 16-DSA solubilized in the same buffer as that of MLKL_2__–154_ sample and the q value of bicelles was the same. A 900 MHz Bruker spectrometer equipped with a cryogenic TXI probe was used to monitor the titration process. Peak intensities at different concentrations of 16-DSA (0, 0.6, 1.2, 1.8, 2.4, 3.0, 3.6, 4.2, 4.8, 5.4 and 6 mM) were recorded by 2D ^1^H-^15^N TROSY-HSQC spectra (3.5 s recover delay). The residue-specific I/I_0_ vs. 16-DSA concentration was plotted.

### 4.7. Molecular Dynamics Simulation

Molecular dynamics (MD) simulations for this protein were carried out in two different conditions, in pure water and membrane, using the academic free Desmond 2020 package (https://www.schrodinger.com/desmond, accessed on 31 March 2021) under the default OPLS-AA 2005 force field [31] with the appropriate number of counter ions to balance the net charge of the system solvated in 150 mM NaCl.

For the first MD simulation in pure water condition, the protein structure (PDB code: 2MSV) [20] was downloaded from the Protein Data Bank (www.rcsb.org, access on 31 December 1971) and embedded in a periodic orthorhombic box (~20 × 20 × 20 Å^3^) containing the SPC water molecules. For the second MD system in the membrane, the membrane localization of the protein (PDB code: 2MSV) and membrane (DMPC) were predicted in the according to the positions reported by Quarato et al. [25]. The second MD system in the membrane was embedded in a periodic orthorhombic box (~40 × 40 × 40 Å^3^) containing the SPC water molecules. The above two complexes were performed with periodic boundary conditions in an NPT ensemble in which the amount of substance (N), pressure (P) and temperature (T) were conserved. Nose-Hoover temperature coupling [32] was used to calculate long-range electrostatic interactions with grid spacing of 0.8 Å. The RESPA integrator [33] was used for reducing calculation time by decreasing the frequency of time-consuming long-range interaction calculations. The real-space part electrostatic and Van der Waals interactions were cut off at 9 Å.

The simulations were conducted by the restrained MD simulation using the default ‘membrane relax’ protocol provided in Desmond by about 2 ns. Upon reaching equilibrium, the system was subject to the unrestrained MD, which was performed by running 100 ns of NPT production simulation without any restriction. The configurations and interval energy were recorded at every 100 ps.

### 4.8. Triton X-114 Assay

The pellets from treated cells were re-suspended in 5× volume of Triton X-114 lysis buffer (20 mM HEPES/pH 7.4, 150 mM NaCl, 2% Triton X-114, and complete protease inhibitor [Roche]) and incubated on ice for 30 min. The cell lysate was centrifuged at 15,000× *g* at 4 °C for 10 min, and then the supernatant was harvested as the detergent soluble fraction. After warming at 30 °C for 3 min, the detergent soluble fraction was centrifuged at 1500× *g* for 5 min at room temperature. The aqueous layer was collected and then re-centrifuged at 1500× *g* for 5 min to remove the contamination from the detergent-enriched layer and saved as the aqueous faction (Aq). The detergent-enriched layer was diluted with a basal buffer (20 mM HEPES/pH 7.4, 150 mM NaCl) to the same volume of the detergent soluble fraction and re-centrifuged at 1500× *g* for 5 min. The washed detergent-enriched layer was diluted with the basal buffer to the same volume as the aqueous faction and saved as the detergent fraction (Det).

## 5. Conclusions

We studied the activation of MLKL-NTR in bicelles using a solution-state NMR technique. The release of an auto-inhibited region (residue 124–153) was observed in the presence of negatively charged phospholipids including cardiolipin and DMPG. Using a molecular dynamics simulation, we probed the intrinsic disordered property of H6 and the stable attachment of the disordered H6 to NBD. The arginine cluster in the C-terminus of the auto-inhibitory region contributed to the plug opening. R to E mutation in the cluster resulted in a compromised activation of NTR in bicelles and inhibited plasma membrane translocation and the necrotic effect of full-length MLKL. Based on these results, we proposed a new role for the arginine cluster in the C-terminus of the auto-inhibitory region during the activation of NTR that pulled the plug away through electrostatic interactions with the acidic headgroups of phospholipids.

## Data Availability

The data presented in this study are available in this article or Supplementary Materials.

## Supplementary Materials

The following are available online, Figure S1: Differences between 2D ^1^H-^15^N TROSY-HSQC spectra of MLKL_2–154_ in DMPC bicelles and in solution, Figure S2: The membrane-association of MLKL_2–154_ promoted by DMPG, Figure S3: The unfolded H6 locked in the inhibitory conformation by electrostatic and hydrophobic interactions, Figure S4: Inverting the charges of the arginine cluster in the C-terminus of the auto-inhibitory region not interfering the overall protein structure.

## Sample Availability

Samples are available from the authors.

## Abbreviations

MLKL: mixed lineage kinase domain-like protein; NBD: N-terminal four-helix bundle domain; RIPK3: Receptor Interacting Serine/Threonine Kinase 3; *At*: *Arabidopsis*; psKD: pseudokinase domain; NTR: N-terminal region; MD: molecular dynamics; 2D: two dimensional; TROSY-HSQC: transverse relaxation optimized spectroscopy and heteronuclear single-quantum coherence; NMR: nuclear magnetic resonance; DMPC: 1,2-dimyristoyl-sn-glycero-3-phosphocholine; DHPC: 1,2-dihexanoyl-sn-glycero-3-phosphocholine; DMPG: 1,2-dimyristoyl-sn-glycero-3-phosphoglycerol; NOE: nuclear Overhauser enhancement; PRE: paramagnetic relaxation enhancement; Gd-DOTA: gadolinium(III) 1,4,7,10-tetraazacyclododecane-1,4,7,10-tetraacetate; 16-DSA: 16-doxyl-stearic acid; SPC: simple point charge; RMSF: Root Mean Square Fluctuation; 3D: three dimensional; NPT: normal pressure and temperature; SSE: Secondary Structure Elements; Aq: aqueous faction; Det: detergent fraction.

## References

[1] Dionísio P.A., Amaral J.D., Rodrigues C.M.P. (2020). Molecular mechanisms of necroptosis and relevance for neurodegenerative diseases. Int. Rev. Cell. Mol. Biol..

[2] Sun L., Wang X. (2014). A new kind of cell suicide: Mechanisms and functions of programmed necrosis. Trends Biochem. Sci..

[3] Sun L., Wang H., Wang Z., He S., Chen S., Liao D., Wang L., Yan J., Liu W., Lei X. (2012). Mixed Lineage Kinase Domain-like Protein Mediates Necrosis Signaling Downstream of RIP3 Kinase. Cell.

[4] Murphy J.M., Czabotar P.E., Hildebrand J.M., Lucet I.S., Zhang J.G., Alvarez-Diaz S., Lewis R., Lalaoui N., Metcalf D., Webb A.I. (2013). The pseudokinase mlkl mediates necroptosis via a molecular switch mechanism. Immunity.

[5] Wang H., Sun L., Su L., Rizo J., Liu L., Wang L.F., Wang F.-S., Wang X. (2014). Mixed Lineage Kinase Domain-like Protein MLKL Causes Necrotic Membrane Disruption upon Phosphorylation by RIP. Mol. Cell.

[6] Samson A.L., Zhang Y., Geoghegan N.D., Gavin X.J., Davies K.A., Mlodzianoski M.J., Whitehead L.W., Frank D., Garnish S.E., FitzGibbon C. (2020). MLKL trafficking and accumulation at the plasma membrane control the kinetics and threshold for necroptosis. Nat. Commun..

[7] Li L., Tong A., Zhang Q., Wei Y., Wei X. (2020). The molecular mechanisms of MLKL-dependent and MLKL-independent necrosis. J. Mol. Cell Biol..

[8] Cai Z., Liu Z.-G. (2018). Detection of MLKL Oligomerization During Programmed Necrosis. Methods Mol. Biol..

[9] Cai Z., Jitkaew S., Zhao J., Chiang H.-C., Choksi S., Liu J., Ward Y., Wu L.-G., Liu Z.-G. (2014). Plasma membrane translocation of trimerized MLKL protein is required for TNF-induced necroptosis. Nat. Cell Biol..

[10] Petrie E.J., Sandow J.J., Jacobsen A.V., Smith B.J., Griffin M.D.W., Lucet I.S., Dai W., Young S.N., Tanzer M.C., Wardak A. (2018). Conformational switching of the pseudokinase domain promotes human mlkl tetramerization and cell death by necroptosis. Nat. Commun..

[11] Huang D., Zheng X., Wang Z.-A., Chen X., He W.-T., Zhang Y., Xu J.-G., Zhao H., Shi W., Wang X. (2017). The MLKL Channel in Necroptosis Is an Octamer Formed by Tetramers in a Dyadic Process. Mol. Cell. Biol..

[12] Liu S., Liu H., Johnston A., Hanna-Addams S., Reynoso E., Xiang Y., Wang Z. (2017). MLKL forms disulfide bond-dependent amyloid-like polymers to induce necroptosis. Proc. Natl. Acad. Sci. USA.

[13] Mahdi L.K., Huang M., Zhang X., Nakano R.T., Kopp L.B., Saur I.M., Jacob F., Kovacova V., Lapin D., Parker J.E. (2020). Discovery of a Family of Mixed Lineage Kinase Domain-like Proteins in Plants and Their Role in Innate Immune Signaling. Cell Host Microbe.

[14] Dondelinger Y., Declercq W., Montessuit S., Roelandt R., Goncalves A., Bruggeman I., Hulpiau P., Weber K., Sehon C.A., Marquis R.W. (2014). Mlkl compromises plasma membrane integrity by binding to phosphatidylinositol phosphates. Cell Rep..

[15] McNamara D.E., Dovey C.M., Hale A.T., Quarato G., Grace C.R., Guibao C.D., Diep J., Nourse A., Cai C.R., Wu H. (2019). Direct Activation of Human MLKL by a Select Repertoire of Inositol Phosphate Metabolites. Cell Chem. Biol..

[16] Dovey C.M., Diep J., Clarke B.P., Hale A.T., McNamara D.E., Guo H., Brown N.W., Cao J.Y., Grace C.R., Gough P.J. (2018). MLKL Requires the Inositol Phosphate Code to Execute Necroptosis. Mol. Cell.

[17] Hildebrand J.M., Tanzer M.C., Lucet I.S., Young S.N., Spall S.K., Sharma P., Pierotti C., Garnier J.-M., Dobson R.C., Webb A.I. (2014). Activation of the pseudokinase MLKL unleashes the four-helix bundle domain to induce membrane localization and necroptotic cell death. Proc. Natl. Acad. Sci. USA.

[18] Tanzer M.C., Matti I., Hildebrand J.M., Young S.N., Wardak A., Tripaydonis A., Petrie E.J., Mildenhall A.L., Vaux D.L., Vince J.E. (2016). Evolutionary divergence of the necroptosis effector MLKL. Cell Death Differ..

[19] Petrie E.J., Birkinshaw R.W., Koide A., Denbaum E., Hildebrand J.M., Garnish S.E., Davies K.A., Sandow J.J., Samson A.L., Gavin X. (2020). Identification of mlkl membrane translocation as a checkpoint in necroptotic cell death using monobodies. Proc. Natl. Acad. Sci. USA.

[20] Su L., Quade B., Wang H., Sun L., Wang X., Rizo J. (2014). A Plug Release Mechanism for Membrane Permeation by MLKL. Structure.

[21] Arnez K.H., Kindlova M., Bokil N.J., Murphy J.M., Sweet M.J., Guncar G. (2015). Analysis of the n-terminal region of human mlkl, as well as two distinct mlkl isoforms, reveals new insights into necroptotic cell death. Biosci. Rep..

[22] Murphy J.M., Lucet I.S., Hildebrand J.M., Tanzer M.C., Young S.N., Sharma P., Lessene G., Alexander W.S., Babon J.J., Silke J. (2014). Insights into the evolution of divergent nucleotide-binding mechanisms among pseudokinases revealed by crystal structures of human and mouse MLKL. Biochem. J..

[23] Davies K.A., Tanzer M.C., Griffin M.D.W., Mok Y.F., Young S.N., Qin R., Petrie E.J., Czabotar P.E., Silke J., Murphy J.M. (2018). The brace helices of mlkl mediate interdomain communication and oligomerisation to regulate cell death by necroptosis. Cell Death Differ..

[24] Rübbelke M., Fiegen D., Bauer M., Binder F., Hamilton J., King J., Thamm S., Nar H., Zeeb M. (2020). Locking mixed-lineage kinase domain-like protein in its auto-inhibited state prevents necroptosis. Proc. Natl. Acad. Sci. USA.

[25] Quarato G., Guy C.S., Grace C.R., Llambi F., Nourse A., Rodriguez D.A., Wakefield R., Frase S., Moldoveanu T., Green D.R. (2016). Sequential engagement of distinct mlkl phosphatidylinositol-binding sites executes necroptosis. Mol. Cell.

[26] Chen W., Zhou Z., Li L., Zhong C.Q., Zheng X., Wu X., Zhang Y., Ma H., Huang D., Li W. (2013). Diverse sequence determinants control human and mouse receptor interacting protein 3 (rip3) and mixed lineage kinase domain-like (mlkl) interaction in necroptotic signaling. J. Biol. Chem..

[27] Chen X., Li W., Ren J., Huang D., He W.T., Song Y., Yang C., Li W., Zheng X., Chen P. (2014). Translocation of mixed lineage kinase domain-like protein to plasma membrane leads to necrotic cell death. Cell Res..

[28] Bansal N., Sciabola S., Bhisetti G. (2019). Understanding allosteric interactions in hMLKL protein that modulate necroptosis and its inhibition. Sci. Rep..

[29] Ma Y., Poole K., Goyette J., Gaus K. (2017). Introducing membrane charge and membrane potential to t cell signaling. Front. Immunol..

[30] Guibao C.D., Petrinjak K., Moldoveanu T. (2019). Uncovering human mixed lineage kinase domain-like activation in necroptosis. Future Med. Chem..

[31] McNamara D.E., Quarato G., Guy C.S., Green D.R., Moldoveanu T. (2018). Characterization of MLKL-mediated Plasma Membrane Rupture in Necroptosis. J. Vis. Exp..

[32] Hoover W.G. (1985). Canonical dynamics: Equilibrium phase-space distributions. Phys. Rev. A Gen. Phys..

[33] Deng Z., Martyna G.J., Klein M.L. (1992). Structure and dynamics of bipolarons in liquid ammonia. Phys. Rev. Lett..

