# Supplementary material for: Positive Charges in the Brace Region Facilitate the Membrane Disruption of MLKL-NTR in Necroptosis
Source: Molecules. 2021 Aug 27;26(17):5194. doi: 10.3390/molecules26175194 (PMC8433767; doi:10.3390/molecules26175194)
Supplement: Supplementary file 1 [file molecules-26-05194-s001.zip › molecules-1317703-supplementary.pdf]

Supporting Information

Keywords: MLKL; brace helix; auto-inhibitory; MD simulation

Authors: Yaqing Yang[1,2], Encheng Xie[1,2], Lingyu Du[1], Yu Yang[1,2], Bin Wu[3], Liming Sun[1,2], Shuqing Wang[4*], Bo OuYang[1,2*]

Author Affiliations

1 State Key Laboratory of Molecular Biology, Shanghai Institute of Biochemistry and Cell Biology, CAS Center for Excellence in Molecular Cell Science, Chinese Academy of Sciences, Shanghai 200031, China; yangyaqing2015@sibcb.ac.cn (Y.Y.); xieencheng2019@sibcb.ac.cn (E.X.); dulingyu@sibcb.ac.cn (L.D.); yangyu2014@sibcb.ac.cn (Y.Y); liming.sun@sibcb.ac.cn (L.S.)

2 University of Chinese Academy of Sciences, Beijing 100049, China

3 National Facility for Protein Science in Shanghai, ZhangJiang lab, Shanghai Advanced Research Institute, Chinese Academy of Sciences, Shanghai 201203, China; bin.wu@sibcb.ac.cn (B.W.)

4 School of Pharmacy, Tianjin Medical University, Tianjin 300070, China

* Correspondence: wangshuqing@tmu.edu.cn (S.W.); ouyang@sibcb.ac.cn (B.O.)

Supplementary figures

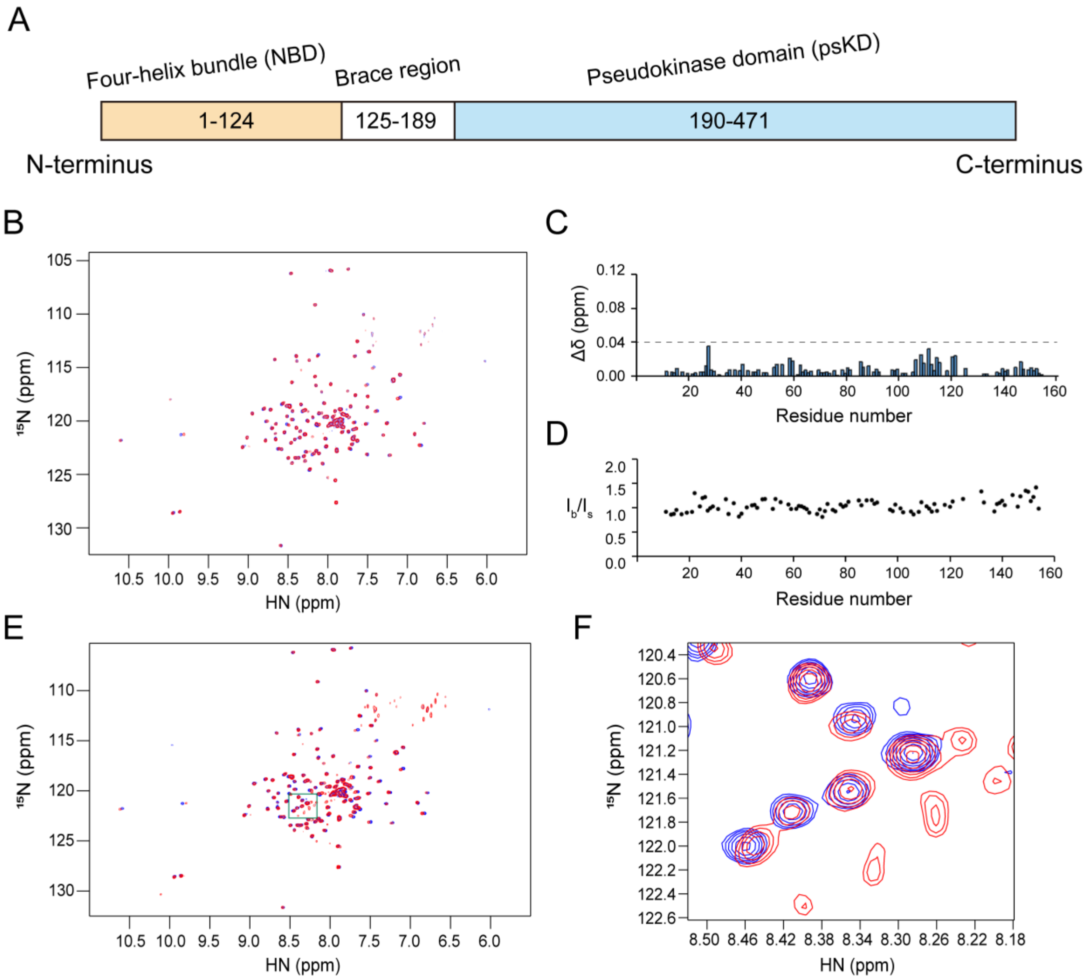

Figure S1. Differences between 2D ^{1}H-^{15}N TROSY-HSQC spectra of MLKL$_{2-154}$ in DMPC bicelles and in solution. (**A**) A linear overview of the domain organization of human MLKL. (**B**) Overlaid 2D ^{1}H-^{15}N TROSY-HSQC spectra of MLKL$_{2-154}$ (0.1 mM) in DMPC bicelles (red) and in solution (blue). (**C**) Chemical shift differences between the two spectra in (B). (**D**) Ratios of residue-specific peak intensities between MLKL$_{2-154}$ in DMPC bicelles (I_b) and in solution (I_s). (**E**) Differences between 2D ^{1}H-^{15}N TROSY-HSQC spectra of MLKL$_{2-154}$ (0.6 mM) in DMPC bicelles (red) and in solution (blue). (**F**) The magnified region shows the same spectral region (squared in green) in the full spectrum in (E).

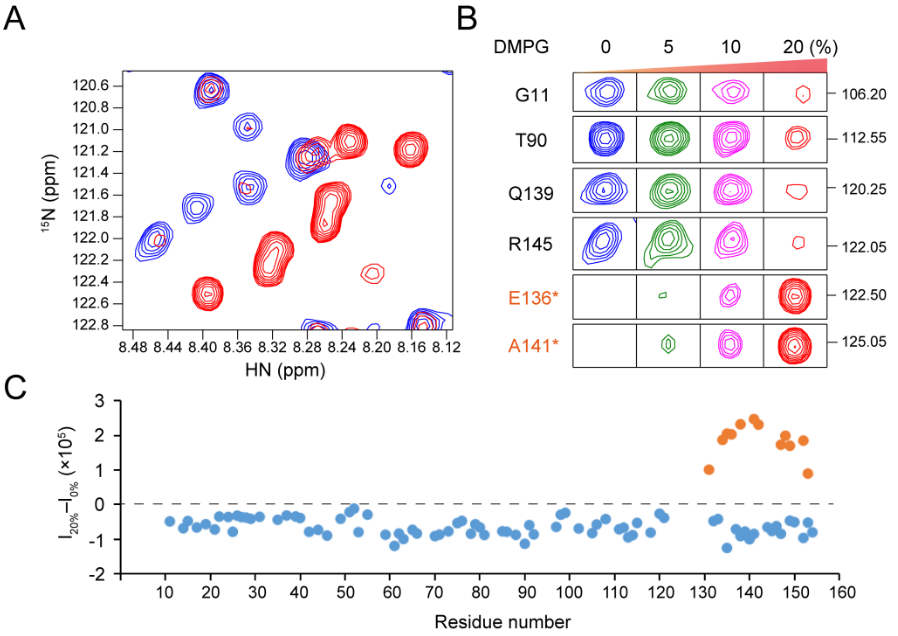

Figure S2. The membrane-association of MLKL$_{2-154}$ promoted by DMPG. (**A**) The overlaid 2D ^{1}H-^{15}N TROSY-HSQC spectra of MLKL$_{2-154}$ in DMPC bicelles with (red) and without (blue) 20% DMPG. (**B**) The peak of six residues of MLKL$_{2-154}$ in DMPC bicelles supplemented with different concentrations of DMPG. The first left panel shows the same spectral region as the right panels for each residue. Panels 2–4 are spectra recorded at increasing concentrations of 5%, 10%, and 20% DMPG. Residue names marked by asterisk and colored in orange are new resonances appeared upon the addition of DMPG. (**C**) Peak intensity changes ($I_{20\%}$–$I_{0\%}$) between MLKL$_{2-154}$ in DMPC bicelles with ($I_{20\%}$) and without ($I_{0\%}$) 20% DMPG. The orange dots denote the newly emerged resonances.

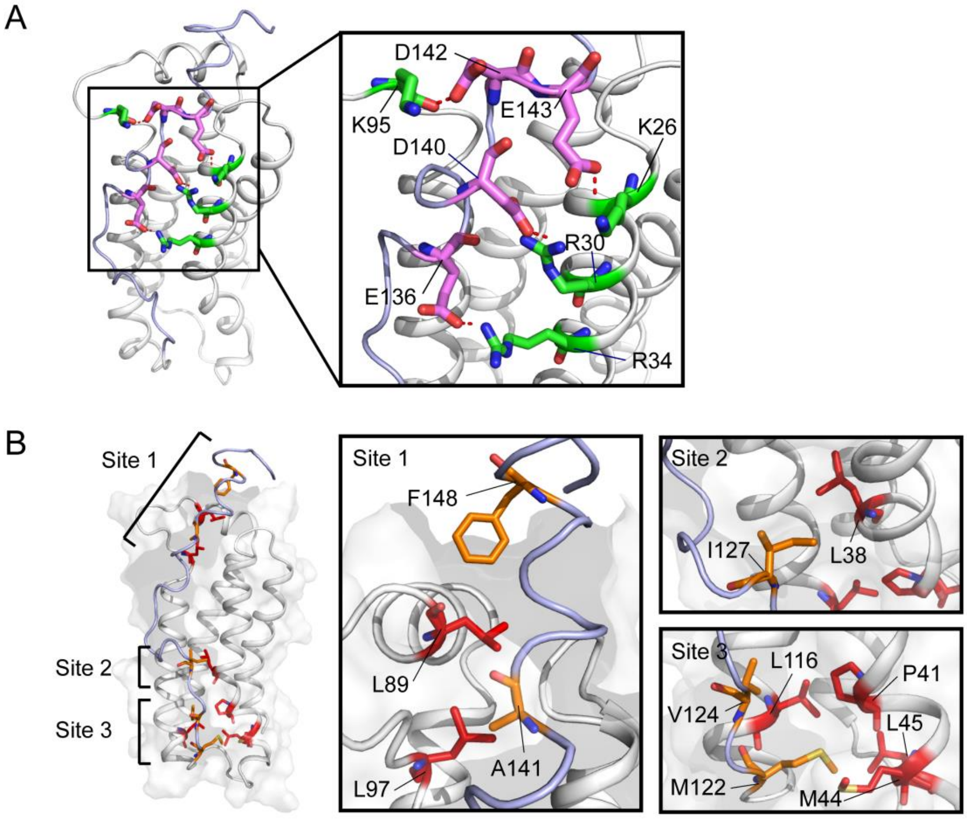

Figure S3. The unfolded H6 locked in the inhibitory conformation by electrostatic and hydrophobic interactions. (**A**) A close-up view of potential electrostatic interactions between the unfolded first brace helix (H6) and the NBD. The residues involved in the interactions are colored magenta in the unfolded H6 and colored green in the NBD respectively. (**B**) Potential hydrophobic interactions between the disordered bracelet and the NBD. The residues involved in the interactions are colored orange in the unfolded bracelet and colored red in the NBD respectively.

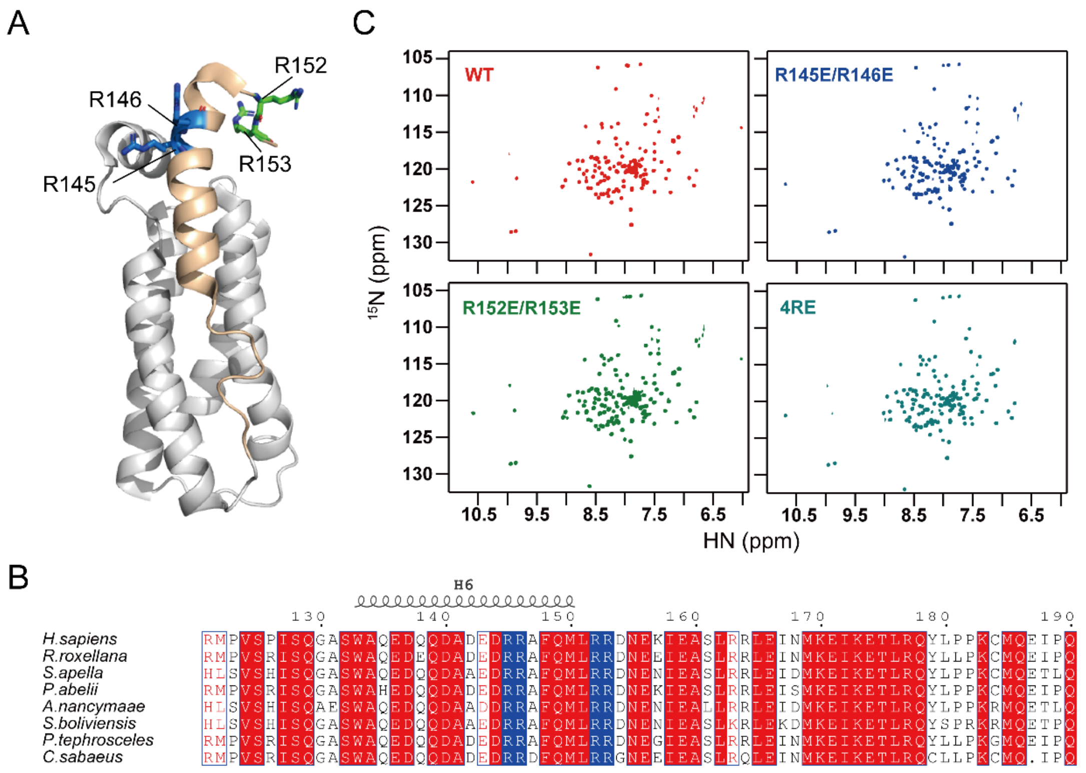

Figure S4. Inverting the charges of the arginine cluster in the C-terminus of the auto-inhibitory region not interfering the overall protein structure. (**A**) Diagram of the solution structure of MLKL$_{2–154}$ (PDB code: 2MSV). The arginine residues in the C-terminus of the auto-inhibitory region (R145, R146, R152 and R153) are shown in sticks with R145, R146 colored marine and R152, R153 colored green. (**B**) Sequence alignment of MLKL$_{121-190}$. A high conservation of the arginine residues is shown among primate species. (**C**) 2D ^{1}H-^{15}N TROSY-HSQC spectra of MLKL$_{2–154}$ WT and mutants (R145E/R146E, R152E/R153E and 4RE) in solution.
